# Eruptive sebaceous hyperplasia induced by oral tacrolimus with good therapeutic response to systemic isotretinoin: a case report^؆^

**DOI:** 10.1016/j.abd.2026.501345

**Published:** 2026-04-22

**Authors:** Guilherme Paiva da Silva, Flávia Regina Ferreira, Fernanda Gonçalves Moya

**Affiliations:** a Dermatology Service, Hospital Municipal Universitário de Taubaté, Universidade de Taubaté, Taubaté, SP, Brazil; b Department of Pathological Anatomy, Hospital do Servidor Público Estadual, São Paulo, SP, Brazil; c Department of Dermatopathology, Faculty of Medicine, Hospital das Clínicas, Universidade de São Paulo, São Paulo, SP, Brazil

Dear Editor,

Sebaceous hyperplasia (SH) is a benign and common condition characterized by an increase in the size of the sebaceous glands that occurs as a result of aging, exposure to ultraviolet radiation, genetic/familial predisposition, or as part of syndromes such as Muir-Torre.^1^, ^2^ Clinically, it presents as small, yellowish or normochromic papules with a central umbilication, usually restricted to the face.^3^

Eruptive sebaceous hyperplasia (ESH) was originally reported in patients receiving solid organ and hematopoietic stem cell transplants using cyclosporine.^3^ The multiplicity of lesions and their occurrence in exposed areas can negatively impact self-esteem, reflecting on interpersonal relationships and the quality of life of these patients. In addition, treatment is challenging due to the number of lesions and the possibility of permanent dyschromias resulting from ablative interventions. Systemic isotretinoin has proven effective in the treatment and control of ESH.^1^, ^4^, ^5^ This report describes a patient with exuberant tacrolimus-induced ESH, with good response to oral isotretinoin monotherapy and control with continuous use of low doses.

A 53-year-old male patient, a kidney transplant recipient, complained of a "skin rash for three to four months." On examination, multiple normochromic asymptomatic papules with central umbilication were present on the face, neck, and anterosuperior thorax (Fig. 1), with abrupt onset months before and progressive worsening. He underwent kidney transplantation four years ago and is currently maintained on tacrolimus monotherapy. He was also an insulin-dependent diabetic. ESH was suspected, and molluscum contagiosum was considered as a differential diagnosis due to immunosuppression, along with eruptive syringoma. An incisional biopsy was performed at two points (face and neck), with histopathological examination showing sebaceous hyperplasia (Fig. 2). Combining the clinical findings, histopathology, and the patient's medication history, a diagnosis of tacrolimus-induced ESH was concluded. This was discussed with the transplant team, and treatment with isotretinoin 20 mg/day was initiated for two months, with significant improvement in the lesions (Fig. 3). Currently, the patient maintains isotretinoin 10 mg, three times a week, without complications or complaints and with maintenance of the therapeutic response.

**Fig. 1:**
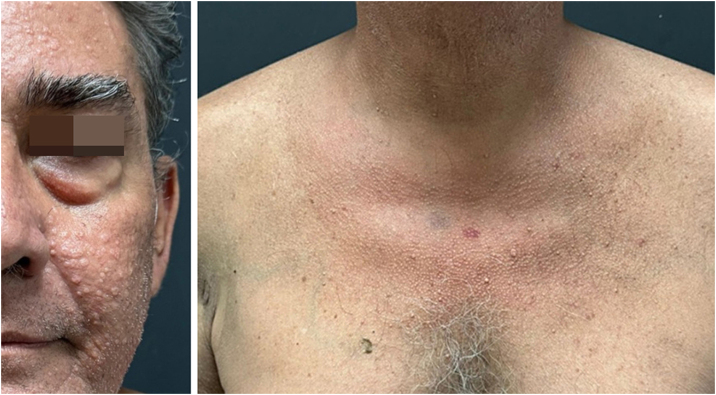
Multiple normochromic papules with central umbilication on the face, neck, and anterosuperior thorax.

**Fig. 2:**
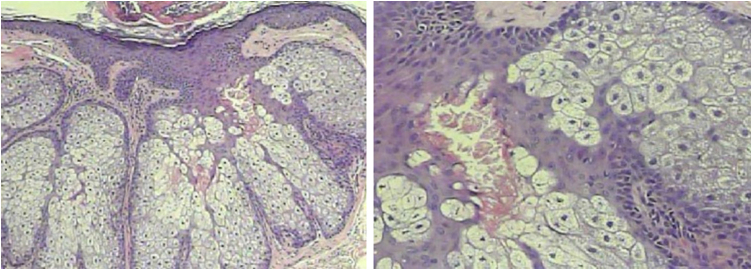
Histopathology showing sebaceous hyperplasia (Hematoxylin & eosin, ×100/400).

**Fig. 3:**
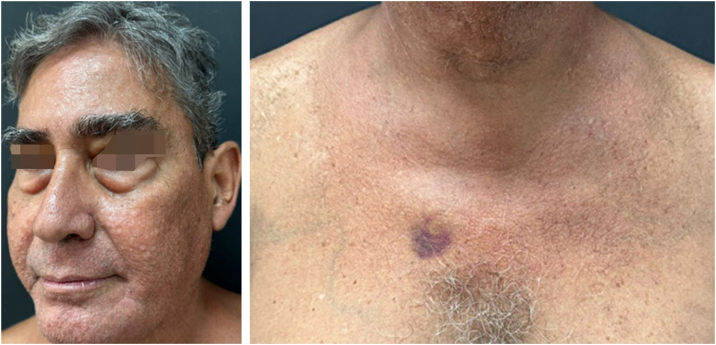
After 2 months of oral isotretinoin (20 mg/day): complete regression of lesions on the trunk and cervical region, with significant improvement in facial lesions.

Currently, ESH seems to be an occurrence practically restricted to adult men, consistent with the present findings, and is strongly associated with immunosuppression induced by medications (cyclosporine, prednisone, mycophenolate mofetil, and tacrolimus) in multiple clinical contexts (kidney disease, allogeneic transplantation, and other systemic inflammatory disorders that require immunosuppression).^6^ Cyclosporine-induced ESH in patients receiving solid organ transplants, such as kidney and heart; and hematopoietic cells is well documented in the literature, showing its association with the development not only of ESH, but also of multiple seborrheic lesions.^7^ As for tacrolimus as a causative agent of ESH, there are few reports, especially when considering its isolated use.^3^

Both cyclosporine and tacrolimus are calcineurin inhibitors, with lipophilic properties that favor their accumulation in the skin.^1^, ^3^, ^6^ This characteristic may contribute to the aberrant proliferation of immature sebocytes, leading to the emergence of ESH.^3^ This effect is believed to result from chronic inhibition of the local immune response and indirect stimulation of sebaceous gland activity.^6^

The aesthetic impact of facial lesions, especially when numerous as in ESH, can negatively affect patients' self-esteem and quality of life.^1^ Furthermore, treatment is challenging due to the multiplicity of lesions, risk of scarring and recurrence.^5^ Ablative procedures, such as electrodissection, cryotherapy, CO_2_ laser and photodynamic therapy, although effective, present a risk of dyschromia and scarring sequelae.^4^

Systemic isotretinoin, on the other hand, acts diffusely and globally, with well-established sebostatic and antiproliferative effects.^2^ In the present case, monotherapy with isotretinoin resulted in significant and rapid clinical improvement, with good tolerance and sustained control with a low dose (10 mg, three times/week).

In conclusion, this report reinforces tacrolimus as a causative agent of ESH, corroborating the scarce reports in the literature, and highlights the efficacy and safety of oral isotretinoin as a therapeutic option, whether in monotherapy or not.

## ORCID ID

Guilherme Paiva da Silva: 0009-0008-5089-9938

Fernanda Gonçalves Moya: 0000-0003-2933-2845

## Financial support

None declared.

## Authors’ contributions

Guilherme Paiva da Silva: Design and planning of the study; drafting and editing of the manuscript; critical review of the literature; photographic documentation and approval of the final version of the manuscript.

Flávia Regina Ferreira: Design and planning of the study; critical review of the manuscript; effective participation in research orientation; intellectual participation in the propaedeutic and/or therapeutic conduct of the studied cases and approval of the final version of the manuscript.

Fernanda G. Moya: Histopathology (analysis and photographic documentation) and approval of the final version of the manuscript.

## Research data availability

Does not apply.

## Conflicts of interest

None declared.
